# The role of environmental sensitivity in the mental health of Syrian refugee children: a multi-level analysis

**DOI:** 10.1038/s41380-024-02573-x

**Published:** 2024-05-03

**Authors:** Andrew K. May, Demelza Smeeth, Fiona McEwen, Elie Karam, Michael J. Rieder, Abdelbaset A. Elzagallaai, Stan van Uum, Francesca Lionetti, Michael Pluess

**Affiliations:** 1 Biological and Experimental Psychology, School of Biological and Behavioural Sciences, Queen Mary University of London, London, UK; 2 Department of War Studies, King’s College London, London, UK; 3 Department of Psychiatry and Clinical Psychology, Balamand University, St Georges Hospital University Medical Center, Institute for Development, Research, Advocacy and Applied Care (IDRAAC), Beirut, Lebanon; 4 Physiology and Pharmacology, Schulich School of Medicine and Dentistry, University of Western Ontario, London, ON Canada; 5 Division of Endocrinology and Metabolism, Schulich School of Medicine and Dentistry, University of Western Ontario, London, ON Canada; 6 Department of Neuroscience, Imaging and Clinical Science, G. d’Annunzio University of Chieti-Pescara, Chieti, Italy; 7 Department of Psychological Sciences, School of Psychology, University of Surrey, Guildford, UK

**Keywords:** Psychology, Physiology, Genetics

## Abstract

Individuals with high environmental sensitivity have nervous systems that are disproportionately receptive to both the protective and imperilling aspects of the environment, suggesting their mental health is strongly context-dependent. However, there have been few consolidated attempts to examine putative markers of sensitivity, across different levels of analysis, within a single cohort of individuals with high-priority mental health needs. Here, we examine psychological (self-report), physiological (hair hormones) and genetic (polygenic scores) markers of sensitivity in a large cohort of 1591 Syrian refugee children across two waves of data. Child-caregiver dyads were recruited from informal tented settlements in Lebanon, and completed a battery of psychological instruments at baseline and follow-up (12 months apart). Univariate and multivariate Bayesian linear mixed models were used to examine a) the interrelationships between markers of sensitivity and b) the ability of sensitivity markers to predict anxiety, depression, post-traumatic stress disorder, and externalising behaviour. Self-reported sensitivity (using the Highly Sensitive Child Scale) significantly predicted a higher burden of all forms of mental illness across both waves, however, there were no significant cross-lagged pathways. Physiological and genetic markers were not stably predictive of self-reported sensitivity, and failed to similarly predict mental health outcomes. The measurement of environmental sensitivity may have significant implications for identifying and treating mental illness, especially amongst vulnerable populations, but clinical utility is currently limited to self-report assessment.

## Introduction

Mental health is increasingly prioritised on the global agenda given its integral role in advancing sustainable development worldwide [1]. Despite efforts to strengthen mental healthcare, guided by the *Comprehensive mental health action plan 2013–2030* [2], mental illness remains a considerable burden affecting over 970 million people [3]. The majority of mental illnesses originate in childhood [4, 5], but often go undiagnosed and untreated as children have the least access to mental healthcare [6]. Amongst four key objectives for promoting mental healthcare, the WHO recommends stronger information systems, evidence, and research [3].

A central goal of contemporary research is identifying risk and protective factors for mental health [7, 8]. Recently, evidence has accrued that highlights environmental sensitivity as a determinant of risk to mental illness [9, 10]. Briefly, environmental sensitivity (ES) theory [11] posits that individuals can be positioned along a spectrum based on how receptive their nervous systems are to both imperilling and protective aspects of the environment. As a meta framework, ES combines three convergent theories on sensitivity which argue along different disciplinary lines, namely *Sensory Processing Sensitivity* [12], *Biological Sensitivity to Context* [13], and *Differential Susceptibility* [14]. Together, these theories postulate that highly sensitive persons (indexed through various proxies of nervous system functioning) are disproportionately more likely to internalise trauma, deeply entrenching its negative effects and exacerbating risks for psychopathology [15]. Conversely, these same persons are more receptive to protective elements, capable of ingraining positive environmental influences (e.g. social support) by virtue of their heightened nervous systems. The reverse is hypothesised for less sensitive people, whose high threshold nervous systems buffer them against the effects of traumatic and supportive environmental influences alike. Put differently, sensitivity is the biologically-embedded filter through which environmental experiences are processed and integrated, which subsequently informs developmental trajectories and evolving personalities. ES theory thus implies that the *felt experience* of environmental factors may be an important component of mental health research and its applications.

True to these theoretical assertions, sensitivity has been empirically linked to health outcomes *for-better-and-for-worse* [16]. Under stressful conditions, highly sensitive persons are particularly prone to depression [17–19], anxiety [20], social phobia [21] and low levels of life satisfaction [22]. But a sensitive disposition has also been associated with improved resilience [23], lowered aggression [24, 25] and fewer problem behaviours [26, 27] where the environmental milieu is supportive. Meanwhile, experimental studies have demonstrated the striking responsiveness of sensitive persons to interventions for depression [28], bullying [29], marital satisfaction [30], and maternal attachment [31], whereas non-sensitive individuals were largely indifferent to these intervention efforts.

While these findings are promising, they remain fragmented, with sensitivity often operationalised through different markers of nervous system functioning. These markers stem from various levels of analysis, including temperament and behaviour, brain structure and neural circuitry, physiological functioning, and genetic variation [16, 32]. Currently, the most widespread measure of environmental sensitivity is the self-report *Highly Sensitive Person* (or Child) scale, which assesses temperament characteristics related to sensory processing and overwhelm [12, 33]. At the physiological level, mean arterial blood pressure [34], blood volume pulse amplitude [35], pubertal timing [36], respiratory sinus arrythmia [26], and cortisol reactivity [37, 38] have all been implicated in interindividual differences in sensitivity. Putative genetic markers of sensitivity include candidate genes such as *DRD4* [27], 5-HTTLPR [39–41], and *COMT* [42], amongst others [43], as well as GWAS-derived polygenic scores (PGS) [15]. Although these markers have, independently, provided evidence supporting ES, there have been few attempts to examine multiple sensitivity markers together, within the same cohort, to discern their degree of correlation, and whether they similarly predict behavioural outcomes such as mental health.

Motivated by this knowledge gap, the aim of the current study was to explore the intercorrelations between mental health and several sensitivity markers across multiple levels of analysis, in a large sample of Syrian refugee children. Previously, we reported the extent of mental health issues in this sample [44], noting that 36.9% of the children met diagnostic criteria for post-traumatic stress disorder (PTSD), 20.1% for depression, 26.9% for externalising disorders, and 47.8% for anxiety. Here, we examined these mental health outcomes in relation to markers of sensitivity covering psychological (self-reported sensitivity), physiological (hair hormones) and genetic (polygenic scores for sensitivity-related traits) levels of analysis. We hypothesised that sensitivity markers would a) meaningfully correlate with each other due to their shared relationship with nervous system functioning, and b) similarly predict mental health outcomes. Additionally, we hypothesised that, on balance, high sensitivity would predict worse mental health in the stressful refugee context.

## Methods and materials

### Study participants

Participants included 1591 Syrian refugee children (and their caregivers) recruited as part of the biological pathways of risk and resilience (BIOPATH) study, detailed elsewhere [44, 45]. Children were 11 years old on average, with an even split in sex (53% female), at the baseline (wave 1) visit. During follow-up (one year after baseline; wave 2), 1000 dyads were successfully recontacted. Importantly, children lost to follow-up were not significantly different on any of the study variables (Supplementary Table S1). Ethical approval was granted by the Institutional Review Board at the University of Balamand/Saint George Hospital University Medical Centre, Lebanon (ref: IRB/O/024-16/1815), the Lebanese National Consultative Committee on Ethics, and the Ministry of Health.

### Instruments

All instruments were translated into Arabic following a published protocol [46], and piloted in the same target population to maximise psychometric performance.

#### Demographic data

Children were asked to supply demographic data on their gender, age, nationality, general health, and health behaviours (e.g. smoking). As covariates for hair hormone analyses, the frequency of hair washing and hair alterations (e.g. chemical straightening, dyes) was noted. Caregivers corroborated responses supplied by children where possible.

#### Mental health outcomes

Childhood depression was measured through the Centre for Epidemiological Studies Depression Scale for Children (CES-DC). The CES-DC is a self-report questionnaire assessing the frequency of symptoms of depression in children and adolescents [47]. The instrument comprises 20 items, scored on a scale from 0 to 3, with higher scores indicating more frequent symptoms. Following pilot testing for the BIOPATH study, the instrument was reduced to 10 items (loading onto a single factor) that were more understandable by refugee children. Internal consistency reliability for the remaining 10 items was high (*α* = 0.88).

To measure PTSD, we used the Child PTSD Symptom Scale (CPSS). The CPSS is a 17-item self-report instrument assessing the severity of DSM-IV PTSD symptoms for use in children aged 8–18 [48]. Item responses are captured on a scale from 0 to 3, with higher scores indicating more frequent symptoms. The CPSS shares a 0.80 correlation with the Child Posttraumatic Stress Reaction Index [49], suggesting strong convergent validity. In the present study, the CPSS demonstrated high internal consistency reliability (Cronbach’s *α* = 0.91).

Self-reported anxiety was captured using the Screen for Child Anxiety Related Emotional Disorders (SCARED) [50]. The SCARED is used to screen for childhood anxiety problems including social phobia, panic disorder, and general and separation anxiety disorder. Although originally 41 items in length, the scale was shortened to 15 items for the BIOPATH study to provide a brief, more general, single-factor measure of anxiety. The selection of which 15 items to retain was informed by factor analysis of pilot data, as well as qualitative feedback from refugee children. Poorly comprehended items, and those pertaining to school anxiety were removed (most refugee children did not have access to schools). The remaining 15 items were scored on a three-point scale (0–2), and attained good internal consistency reliability across both waves (*α* = 0.82).

As a supplement to child-report mental health issues, caregivers were asked to gauge their child’s mental wellbeing through the Strengths and Difficulties Questionnaire (SDQ) [51, 52]. The SDQ is a 25-item instrument designed to capture psychological attributes of children aged between 3 and 16 years, and has been previously translated and used in research on Syrian refugees [53]. Items can be divided between five or three subscales [54]. We focused on the externalising (10 item) subscale, which was supplemented with a further set of 12 items aligning to the DSM-V criteria for conduct disorder and oppositional defiant disorder [45]. Items were rated on a three-point scale (0–2), with higher scores indicating more frequent behaviour (three items were reverse-scored). The combined scale of 22 items attained acceptable internal consistency reliability (*α* = 0.65 at wave 1 and 0.72 at wave 2).

#### Exposure to war

The War Events Questionnaire (WEQ) was designed by the Institute for Development, Research, Advocacy and Applied Care (IDRAAC) to measure the degree of civilian exposure to war events, specifically for use in Lebanon [55]. The instrument comprises 25 items which assess experiences of explicit war events such as violence, injury or kidnapping and was administered to both the child and caregiver. Because self-report may be less reliable in younger children [56], child and caregiver responses were combined such that if either one reported that the child experienced an event, the event was considered to have occurred. The WEQ has been used amongst adult Syrian refugees previously, where it performed adequately [57]. In our sample, internal consistency reliability for the instrument was high across both waves (Cronbach’s *α* = 0.88).

#### Environmental sensitivity

##### Self-report

The Highly Sensitive Child scale is a 12-item self-report scale that gauges levels of sensory processing sensitivity in children and adolescents [12, 33]. The scale comprises three factors, namely Ease of Excitation (EOE), Low Sensory Threshold (LST), and Aesthetic Sensitivity (AES). Test-retest reliability for the instrument across 15 days was good (*r* = 0.68), as was internal consistency reliability (*α* = 0.78) [33]. Reliability was reduced, but adequate in the study sample (*α* = 0.73 at wave 1 and 0.63 at wave 2).

##### Hair hormones

Levels of hair cortisol were measured as described previously [58], with testosterone and dehydroepiandrosterone (DHEA) measured following identical procedures. Hair hormone values were log transformed to improve their distribution, and then corrected for batch effects by subtracting the mean value per batch from each of the readings within that batch.

##### Genetic data

Saliva samples were collected using Isohelix GeneFix collection kits and genomic DNA was extracted. Genotyping was performed on the Illumina Infinium Global Screening Array (comprising 650,181 markers, with coordinates based on GRCh37). Quality control was conducted using PLINK version 1.90 [59, 60]. Twenty-eight samples were removed due to discrepancies between pedigree and genotyped sex, 68 samples were removed for an individual genotype missing rate >2%, and six samples were removed for outlying heterozygosity rates (F > 0.2). We did not remove individuals based on identity-by-descent due to known consanguinity amongst Syrian refugees [61]. Variants with a missing rate >3%, a minor allele frequency less than 1%, and/or a Hardy–Weinberg equilibrium *p*-value < 1 ×10^-6^ were excluded, leaving 420,463 markers.

Imputation of additional genotypes was performed using the Haplotype Reference Consortium r1.1 2016 panel (GRCh37/hg19; 97.77% overlap) and polygenic scores for neuroticism/sensitivity (Supplementary Table S2) were calculated (using PGS Catalogue v20230119, https://www.pgscatalog.org/) by means of the Michigan Imputation Server’s Genotype Imputation and Polygenic Scores service (Beta version 1.7.1; https://imputationserver.sph.umich.edu). Imputed variants with an Rsq quality score >0.3 and a minor allele frequency >0.01 were retained.

We calculated additional polygenic scores using summary statistics for traits that proxy sensitivity. The first of these summary statistics were based on a GWAS of (discordant) emotional symptoms in monozygotic twins [15], originally performed for the purpose of constructing an ES PGS. The second set of summary statistics pertained to the construct of sensitivity to environmental stress and adversity (SESA) [62]. SESA is a genetically distinct cluster of neuroticism, along with “depressed affect” and “worry”, each defined by four items from the 12-item version of the Eysenck Personality Questionnaire [63]. Two further sets of summary statistics were obtained from meta-analyses by the Genetics of Personality Consortium (https://tweelingenregister.vu.nl/gpc) for neuroticism [64] and extraversion (harmonised across 30 cohorts) [65, 66], given documented associations between these personality traits and sensitivity [67]. Lastly, we created a sensitivity PGS based on candidate genes by additively tallying together 13 SNPs (0, 1 or 2 alleles) previously linked to higher sensitivity (Supplementary Table S3), as has been similarly performed elsewhere [30, 68]. Summary statistics were prepared and quality controlled according to recommended guidelines, and final scores were z-standardised [69]. Scores were calculated using a clumping and thresholding approach implemented by PRSice [70], while controlling for the first ten principal components.

### Data analysis

Descriptive summaries were prepared and bivariate analyses were conducted using the *gtsummary* package [71]. Intraclass correlation coefficients (ICCs) were calculated for those variables measured across waves. Zero-order correlations were tabulated using the *datscience* package [72] and network diagrams were prepared using the *bootnet* package [73].

An association test between self-reported sensitivity (both as a single construct and as three factors) and other potential predictors of sensitivity was conducted using two-level univariate and multivariate Bayesian linear mixed models (LMMs) via the *brms* package [74]. The first ten principal components were added to control for ancestry. To investigate the relationship between markers of sensitivity and mental health, separate models were fitted for self-reported mental health and caregiver-reported externalising behaviour. To account for the relatedness between PTSD, anxiety, and depression, each measure was scaled, and a single multivariate LMM was fitted. For all models, four chains were used, with 4000 sampling iterations per chain. $\hat{R}$ values for all predictors were 1.00 and bulk and tail effective sample sizes were sufficiently large. Cross-lagged panel models assessing self-reported sensitivity and mental health outcomes across both waves were conducted using *lavaan* [75].

All analyses were conducted using R (version 4.2.2) [76] and RStudio (version 2023.03.1 + 446) [77]. Tables were prepared using the *flextable* package [78].

## Results

**Fig. 1:**
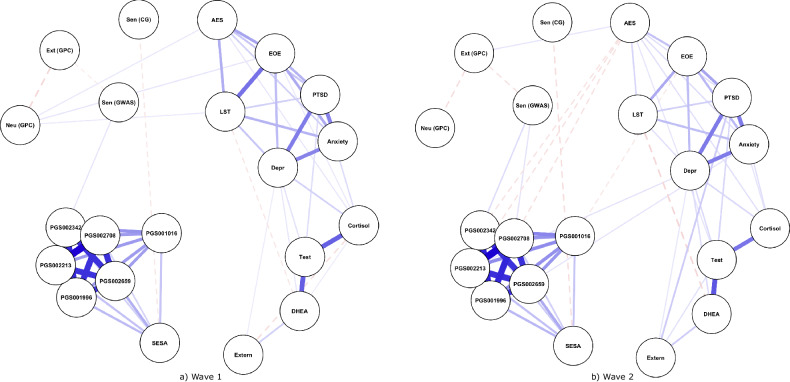
Zero-order correlation network diagrams for each wave of data. Solid edges indicate positive correlations whilst dashed edges represent negative correlations. Edge thickness indicates the strength of correlation. AES aesthetic sensitivity, CG candidate gene, Depr depression, DHEA dehydroepiandrosterone, EOE ease of excitation, Ext extraversion, Extern externalising, GPC Genetics of Personality Consortium, GWAS genome-wide association study, Neu neuroticism, Sen sensitivity, SESA sensitivity to environmental stress and adversity.

Participant demographics, average item scores, and hair hormone variables are summarised in Table 1 (more detailed descriptive overviews appear in Supplementary Tables S4–6). There were several significant zero-order correlations between sensitivity markers (Fig. 1 and Supplementary Table S7). The GPC-derived PGS for neuroticism was positively correlated with total HSC, EOE, LST and AES scores. Cortisol had similar positive correlations, although the relationship to LST did not reach significance. There were significant negative correlations between DHEA and LST, as well as EOE and the candidate gene PGS for sensitivity. None of the PGSs meaningfully correlated with hair hormones. ICCs for variables measured at both waves are shown in Supplementary Table S8. Notably, HSC scores evinced the lowest variability *between* participants for the two time-points (ICC = 0.09), whereas hormone levels varied substantially (0.44-0.63).

**Table 1:** Descriptive statistics for the study sample.

	Wave 1	Wave 2
Characteristic	*N* = 1 591^a^	*N* = 1000^a^
Child age	11.37 (2.42)	12.18 (2.37)
BMI	18.0 (3.6)	18.5 (3.5)
War exposure	9.3 (5.3)	9.3 (5.3)
HSC (total)	5.04 (1.02)	4.77 (0.96)
Ease of excitation	4.74 (1.39)	4.39 (1.43)
Low sensory threshold	5.00 (1.62)	4.84 (1.65)
Aesthetic sensitivity	5.43 (1.10)	5.21 (1.17)
PTSD	0.92 (0.72)	0.63 (0.77)
Depression	0.82 (0.70)	0.64 (0.69)
Anxiety	1.03 (0.45)	0.89 (0.47)
Externalising behaviour (caregiver report)	0.65 (0.22)	0.65 (0.25)
Log cortisol (corrected)	0.00 (0.44)	0.00 (0.41)
Log testosterone (corrected)	0.00 (0.54)	0.00 (0.42)
Log DHEA (corrected)	0.00 (0.35)	0.00 (0.29)
Sex		
*Females*	836 (53%)	535 (54%)
*Males*	755 (47%)	465 (46%)
Ever smoked		
*No*	1569 (99%)	987 (99%)
*Yes*	20 (1%)	11 (1%)
Frequent hair alterations		
*No*	1156 (73%)	760 (76%)
*Yes*	433 (27%)	238 (24%)
Frequency of hair washing		
*2–4 times per week*	969 (61%)	555 (56%)
*5–7 times per week*	496 (31%)	399 (40%)
*Once per week*	124 (7.8%)	44 (4%)

^a^Mean (SD); *n* (%).

To assess which predictor variables remained associated with self-reported sensitivity across both waves whilst controlling for confounders, we tested for association using Bayesian univariate and multivariate linear mixed models (with noninformative priors). As covariates, we included BMI, smoking behaviour, hair alterations and washing frequency (which may affect hair hormone levels), and the first ten principal components to control for genetic ancestry (for brevity, these covariates are not tabulated). Model results for sensitivity as a single construct are summarised in Table 2 (see Supplementary Table S9 for sensitivity subscales). Sensitivity significantly declined from baseline to follow-up, but females scored higher than males whether sensitivity was regarded as a total score or three subscales. Amongst all sensitivity markers, only a PGS for extraversion was credibly linked to self-reported sensitivity, driven by its association with AES. None of the hair hormones remained predictive of sensitivity levels, despite significant zero-order correlations described above.

**Table 2:** Regression model coefficients for predictors of self-reported sensitivity.

Coefficient	Estimate	Error	Lower 95% CI	Upper 95% CI
Intercept	4.243	0.298	**3.662**	**4.842**
Wave	−0.361	0.051	**−0.461**	**−0.261**
Sex (female)	0.396	0.069	**0.256**	**0.530**
Age	0.006	0.014	−0.021	0.033
War exposure	0.013	0.005	**0.003**	**0.022**
Log cortisol	−0.002	0.078	−0.152	0.151
Log testosterone	0.092	0.082	−0.068	0.257
Log DHEA	0.068	0.108	−0.144	0.281
PGS001996	−0.047	0.055	−0.154	0.061
PGS002213	−0.024	0.071	−0.163	0.114
PGS002342	−0.034	0.088	−0.204	0.135
PGS002659	0.005	0.037	−0.065	0.077
PGS002708	0.060	0.080	−0.098	0.216
PGS001016	−0.019	0.027	−0.074	0.033
SESA	0.015	0.027	−0.037	0.066
Sensitivity (GWAS)	−0.022	0.026	−0.074	0.030
Neuroticism (GPC)	0.030	0.026	−0.021	0.081
Extraversion (GPC)	0.056	0.026	**0.006**	**0.106**
Sensitivity (CG)	0.005	0.026	−0.044	0.055

Bolded intervals are those not including 0.

*CI* Credible Interval, *CG* candidate gene, *SESA* Sensitivity to environmental stress and adversity, *GPC* Genetics of Personality Consortium, *PGS* polygenic score.

We then investigated the relationship between mental health and sensitivity markers. Considering there tends to be poor correlation between self- and parent-report childhood mental health [4], two separate Bayesian models were fitted (with noninformative priors). Results of these models are displayed in Tables 3 and 4 (see Supplementary Tables S10 and 11 for sensitivity subscales). Higher self-reported sensitivity was significantly associated with all forms of self-report mental health. This was mostly driven by EOE, which was also implicated in all mental illnesses. LST played a significant role in anxiety and depression, whilst AES appeared to predict anxiety, although the lower bound of the credible interval included 0. Few other sensitivity markers predicted mental health outcomes. Elevated DHEA appeared to predict more symptoms of depression and anxiety. Meanwhile, a PGS for neuroticism (PGS002659) negatively associated with PTSD symptoms. In general, females were more prone to anxiety and depression, but not PTSD, and older children were less anxious, but more likely to present with depression. Expectedly, higher war exposure was a significant predictor of mental illness, regardless of measure. For caregiver-report externalising behaviour, higher sensitivity (total score), PGS0019966, and cortisol levels were marginally linked to externalising problems.

**Table 3:** Association of sensitivity markers with self-reported mental health outcomes.

Coefficient	Estimate	Error	Lower 95% CI	Upper 95% CI
**Anxiety**				
Intercept	−0.773	0.321	**−1.403**	**−0.142**
Wave	0.129	0.052	**0.027**	**0.228**
Sex (female)	0.316	0.070	**0.176**	**0.453**
HSC	0.224	0.026	**0.173**	**0.277**
Age	−0.042	0.014	**−0.068**	**−0.015**
War exposure	0.015	0.005	**0.005**	**0.024**
Log cortisol	0.142	0.078	−0.010	0.295
Log testosterone	0.011	0.082	−0.150	0.166
Log DHEA	0.219	0.111	**0.004**	**0.438**
PGS001996	0.039	0.055	−0.068	0.146
PGS002213	−0.027	0.070	−0.162	0.109
PGS002342	0.070	0.087	−0.103	0.239
PGS002659	−0.019	0.037	−0.091	0.055
PGS002708	−0.000	0.080	−0.155	0.156
PGS001016	−0.010	0.028	−0.064	0.044
SESA	−0.007	0.027	−0.060	0.046
Sensitivity (GWAS)	−0.002	0.026	−0.054	0.049
Neuroticism (GPC)	0.016	0.027	−0.035	0.069
Extraversion (GPC)	0.011	0.026	−0.041	0.062
Sensitivity (CG)	−0.037	0.026	−0.088	0.013
**PTSD**				
Intercept	−0.847	0.323	**−1.485**	**−0.214**
Wave	0.026	0.053	−0.079	0.130
Sex (female)	−0.023	0.069	−0.157	0.114
HSC	0.206	0.027	**0.154**	**0.257**
Age	0.026	0.014	−0.000	0.053
War exposure	0.026	0.005	**0.016**	**0.036**
Log cortisol	0.072	0.077	−0.080	0.223
Log testosterone	0.104	0.081	−0.058	0.264
Log DHEA	0.050	0.110	−0.165	0.263
PGS001996	0.062	0.056	−0.047	0.173
PGS002213	0.041	0.071	−0.096	0.179
PGS002342	0.124	0.089	−0.049	0.294
PGS002659	−0.097	0.038	**−0.170**	**−0.023**
PGS002708	−0.112	0.081	−0.270	0.048
PGS001016	−0.039	0.028	−0.094	0.015
SESA	0.024	0.028	−0.031	0.078
Sensitivity (GWAS)	−0.033	0.027	−0.085	0.019
Neuroticism (GPC)	0.045	0.026	−0.005	0.097
Extraversion (GPC)	−0.015	0.026	−0.065	0.035
Sensitivity (CG)	−0.007	0.026	−0.057	0.045
**Depression**				
Intercept	−1.508	0.322	**−2.147**	**−0.875**
Wave	0.053	0.050	−0.042	0.147
Sex (female)	0.156	0.070	**0.016**	**0.294**
HSC	0.163	0.026	**0.111**	**0.215**
Age	0.055	0.014	**0.028**	**0.082**
War exposure	0.022	0.005	**0.012**	**0.032**
Log cortisol	0.101	0.078	−0.051	0.254
Log testosterone	−0.123	0.080	−0.280	0.034
Log DHEA	0.367	0.110	**0.151**	**0.585**
PGS001996	−0.022	0.056	−0.132	0.087
PGS002213	0.052	0.070	−0.085	0.191
PGS002342	0.084	0.090	−0.090	0.264
PGS002659	−0.022	0.037	−0.096	0.052
PGS002708	−0.021	0.083	−0.187	0.143
PGS001016	0.006	0.028	−0.048	0.059
SESA	−0.007	0.028	−0.062	0.047
Sensitivity (GWAS)	0.010	0.027	−0.043	0.064
Neuroticism (GPC)	0.019	0.027	−0.032	0.070
Extraversion (GPC)	−0.036	0.026	−0.087	0.015
Sensitivity (CG)	0.008	0.026	−0.045	0.059

Bolded intervals are those not including 0.

*CI* Credible Interval, *CG* candidate gene, *SESA* Sensitivity to environmental stress and adversity, *GPC* Genetics of Personality Consortium, *PGS* polygenic score.

**Table 4:** Association of sensitivity markers with caregiver-reported externalising behaviour.

Coefficient	Estimate	Error	Lower 95% CI	Upper 95% CI
Intercept	0.795	0.077	**0.644**	**0.941**
Wave	0.021	0.012	−0.002	0.044
Sex (female)	−0.106	0.016	**−0.138**	**−0.073**
HSC	0.011	0.006	−0.001	0.023
Age	−0.013	0.003	**−0.019**	**−0.006**
War exposure	0.004	0.001	**0.002**	**0.007**
Log cortisol	0.041	0.018	**0.006**	**0.076**
Log testosterone	0.003	0.019	−0.033	0.040
Log DHEA	−0.007	0.026	−0.057	0.044
PGS001996	0.030	0.013	**0.005**	**0.056**
PGS002213	−0.018	0.017	−0.051	0.014
PGS002342	0.020	0.021	−0.022	0.061
PGS002659	−0.010	0.009	−0.027	0.008
PGS002708	−0.014	0.019	−0.052	0.024
PGS001016	−0.002	0.007	−0.015	0.011
SESA	0.003	0.007	−0.010	0.015
Sensitivity (GWAS)	−0.000	0.006	−0.013	0.012
Neuroticism (GPC)	0.007	0.006	−0.006	0.019
Extraversion (GPC)	−0.011	0.006	−0.023	0.001
Sensitivity (CG)	0.001	0.006	−0.011	0.013

Bolded intervals are those not including 0.

*CI* Credible Interval, *CG* candidate gene, *SESA* Sensitivity to environmental stress and adversity, *GPC* Genetics of Personality Consortium, *PGS* polygenic score.

**Fig. 2:**
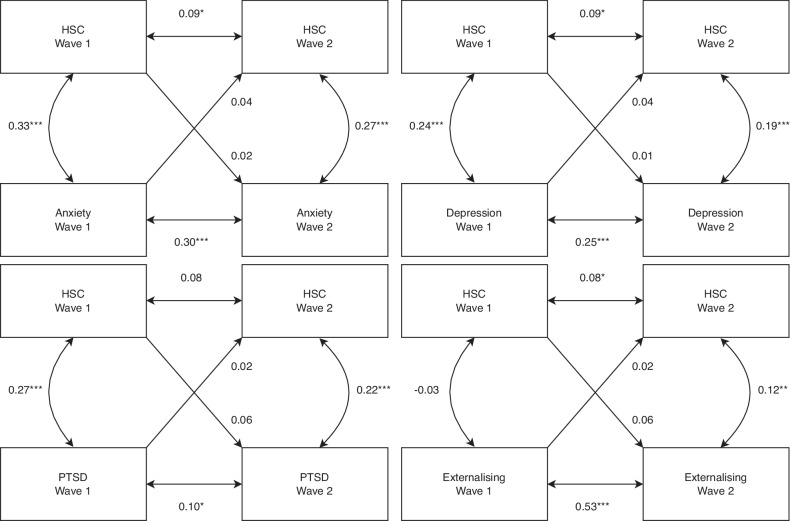
Cross-lagged panel models*.* The Highly Sensitive Child (HSC) scale and self-reported mental health outcomes shared no significant cross-lagged pathways across waves.

As a final investigation, we fitted cross-lagged panel models using self-reported sensitivity and each of the mental health instruments. Sex, age and war exposure were controlled for, and residual measurement invariance across waves was assumed. Notably, we found no significant cross-lagged pathways (Fig. 2).

## Discussion

Environmental sensitivity significantly informs the effects of both trauma and support on children and adults, marking the trait as an important interindividual difference with practical implications for mental health [79–81]. Screening individuals for differing levels of sensitivity may be useful in personalising mental health care [82], but putative indices for sensitivity are numerous, and are spread across different levels of analysis [16, 32], with few attempts to consolidate these markers. In the current study, we examined several sensitivity markers together, investigating both their interrelationships and their correlation to mental health in Syrian refugee children. Overall, our original hypotheses were partially supported.

Within a single cohort, we analysed markers of sensitivity across psychological, physiological, and genetic levels of analysis. In line with our first hypothesis, zero-order correlations suggested significant positive relationships between a subset of these markers, namely self-reported sensitivity, cortisol levels, and genetic disposition to neuroticism. Although we incorporated a range of PGSs, only one PGS, calculated from the summary statistics of the Genetics of Personality Consortium’s GWAS of neuroticism (the largest such GWAS to date) [64], correlated to sensitivity. The greater power of this GWAS likely resulted in a strong set of association signals that remained informative for Syrian refugees. That we generated this PGS whilst controlling for the principal components of genetic ancestry in our sample possibly improved its applicability over others for neuroticism.

When controlling for covariates, however, our association test across two waves of data (Table 2) did not support strong relationships between self-report and other sensitivity markers. We attribute this lack of association to several probable reasons. Firstly, based on the ICCs (Supplementary Table S8), self-reported sensitivity evinced proportionally more *within-person* than *between-person* (0.09) variance, suggesting lower trait stability over one year than has been previously documented [83]. Importantly, the stability of sensitivity in children appears to fluctuate in relation to the harshness of the environment [83] - an effect possibly complicated by the unpredictable refugee context - making it notably more challenging to identify stable associations. In future analyses, possible causes underpinning this high *within-person* variability should be investigated. Secondly, hair hormone levels demonstrated substantial *between-person* variability from baseline to follow-up, likely due to the numerous factors that can impact hormone secretion [84], and given the speculated potential of hair hormones to reflect effects of chronic and acute stressors [85]. Moreover, stress hormone secretion levels are not linearly related to stress, exemplified by the phenomenon of cortisol blunting [86]. As a sex hormone, testosterone levels would have been in the midst of large fluctuations given the proximity of puberty in our age range of refugee children. With the capacity for such wide variability, hormone levels might only be good point estimates of nervous system functioning (and therefore, sensitivity), rather than long term indicators, explaining our lack association findings. However, studies with more waves are needed to confirm this reasoning. Thirdly, due to the distance between genotype and phenotype [87], PGSs explain minimal amounts of phenotypic variation, and significant debate surrounds their practical utility [88, 89]. Because PGSs total the narrow-sense SNP-based heritability of traits, with no acknowledgement of dominant, multiplicative or other interactive effects, they are particularly weak for personality-level phenotypes [89, 90]. While we did detect a possible role for an extraversion PGS to predict high sensitivity levels, this was driven by an association to AES; the subscale with lower reliability due to risks for social desirability bias [91]. A better strategy may be to explore genetic variation influencing endophenotypes of sensitivity [92], once reliable examples have been confirmed.

When exploring the relationships between sensitivity markers and mental health, we found self-reported high sensitivity to predict increased mental illness as hypothesised. This was expected, given that sensitivity has been previously linked to low resilience in this sample [9], as well as a multitude of mental illnesses in other studies. Moreover, these findings support the theoretical framework of ES, providing further evidence that highly sensitive children are disproportionately prone to mental illness under stressful circumstances. Rather than being a causative risk factor for psychopathology, however, it may also be possible that heightened environmental sensitivity, to some degree, reflects a conditional adaptation by traumatised children who are afforded fitness-enhancing hypervigilance at the expense of mental health burdens [93], which might be particularly evident amongst refugee children.

Against our expectations, only one other putative proxy of sensitivity emerged as a predictor of mental health. Specifically, we noted that elevated DHEA levels were associated with higher anxiety and depression. Recently, a meta-analysis of the hormone [94] concluded that DHEA levels (measured in saliva and blood) increased following acute mental stress, particularly in females and young individuals. Whether these conclusions are generalisable to hair hormone levels in chronically mentally stressed children is debatable, but our results should encourage further exploration of DHEA as a biomarker for mental illness. As an abundantly circulating hormone with numerous biological effects [95], DHEA plausibly reflects nervous system functioning, although it hasn’t been directly indicated as a marker of sensitivity. While DHEA and LST shared a significant negative zero-order correlation in our sample, we found no other support for a correlation between sensitivity and DHEA, despite the hormone similarly predicting mental health outcomes compared to self-report sensitivity. This is possibly further testament to the arguments above regarding hormone variability and stable prediction of sensitivity.

We did not detect any significant cross-lagged paths between sensitivity and mental health across both waves of data (Fig. 2). This suggests that self-reported sensitivity is not merely a proxy measurement of mental illness, nor does mental illness influence perceived sensitivity, corroborating assertions that sensitivity is an independent trait with outcomes that are contingent on the environmental context. However, the autoregressive path between self-reported sensitivity at waves 1 and 2 was minimally significant (±0.08), further highlighting high *within-person* variance in sensitivity which may have obscured potential cross-lagged effects. Additionally, we did not have sufficient waves of data to afford more robust random intercepts cross lagged panel models [96], thus our current models should be treated as exploratory.

Few other studies have considered ES in the context of child refugee mental health outcomes. Karam and colleagues [10] noted that highly sensitive children, without a previous history of significant adversities, were most susceptible to PTSD following war exposure. Where war exposure was not amongst the first of the child’s adversities, high sensitivity did not predict susceptibility to PTSD. Together with our findings, it is further apparent that ES theory has translational value to mental health care, both for refugees and more generally [82, 97]. Most importantly, screening for high sensitivity may aid clinicians in earmarking children not only in particular need of support, but also most likely to benefit from treatment [28]. More research is needed in order to determine the specific clinical implications for the treatment of children at different levels of environmental sensitivity (both high and low), but given the typical need of traumatised children for emotional and social support besides information about their particular situation [98], care regimens that simultaneously promote adaptive cognitive and behavioural coping strategies may be especially beneficial for highly sensitive children. However, measuring childhood sensitivity seems best achieved through self- or other-reported approaches, which capture the full construct of ES. Whilst the exploration of various biological markers for sensitivity remains an interesting research avenue that may yield more plausible applications in future, such markers may have little current relevance to clinical practice.

### Strengths and limitations

Particular strengths of our study include its novelty both in design and refugee setting. Ours is amongst the first studies to investigate ES markers from multiple levels of analysis in a single cohort, across two waves of data. With a focus on refugee children of non-European ancestry, our study was not only more reflective of understudied populations, but also less prone to the disadvantages of typical voluntary studies, which attract highly educated adults of fairly robust mental health [4].

Regarding limitations, since refugees are a heterogeneous group stemming from an eclectic set of circumstances, the generalisability of our findings is limited [99]. Also, we did not have a suitable control/comparison group to better contextualise our findings [100]. Because our sample differed in ancestry to the base samples used to inform pre-existing PGSs (from the PGS catalogue), or those used to generate GWAS summary statistics (all of which were predominantly European ancestry), this presumably created issues with score deflation, which is magnified for traits with strong GxE interactions, such as sensitivity [16, 69]. Furthermore, there are currently no large-scale GWASs on the trait of sensitivity, which forced us to generate PGSs based on associated traits assumed to have shared genetic underpinnings. The clumping and thresholding approach used in our score generation is prone to overfitting [69], which can only be guarded against by optimising PGSs in independent samples that we do not currently have.

## Conclusion

Our study indicates that self-report highly sensitive refugee children are significantly prone to mental illness. These findings further support ES theory, which predicts worse outcomes in stressful contexts for individuals with highly reactive nervous systems, and encourage future mental health research and care efforts to carefully consider sensitivity differences. However, we did not find substantial correlations between different markers of sensitivity identified to-date, suggesting that more work is required to expand the objective assessment of ES, so that the theoretical implications can be better translated to practice.

## Supplementary information

Supplementary Material

## Data Availability

To protect the privacy of our vulnerable refugee participants, data are available on request to the corresponding author.

## Code availability

Code is available on request to the corresponding author.
