# Supplementary material for: The role of environmental sensitivity in the mental health of Syrian refugee children: a multi-level analysis
Source: Mol Psychiatry. 2024 May 3;29(10):3170–9. doi: 10.1038/s41380-024-02573-x (PMC11449786; doi:10.1038/s41380-024-02573-x)
Supplement: Supplementary file 1 — Supplementary Material [file 41380_2024_2573_MOESM1_ESM.docx]

**Supplementary Information**

**Sensitivity analyses**

To gauge the robustness of our findings, we explored several sensitivity analyses (not shown). For one such analysis, we imputed five complete datasets and attempted to fit the same linear mixed models. However, these models were computationally intensive and did not converge well, resulting in problematic $\hat{R}$ values that undermined any interpretation of the resulting posterior distributions. Twisk (2013) notes that multiple imputation can lead to instability in mixed model results, even when using up to 50 imputed datasets. In another attempt to try boost study power by reducing data missingness, we omitted the covariate with the most missing data (BMI). This allowed a further ~300 individuals to be incorporated in the linear mixed models who were otherwise removed via listwise deletion. Under these modelling conditions, the PGS for neuroticism derived from the GPC GWAS emerged as a significant predictor of sensitivity (specifically EOE), which is understandable given the significant zero-order correlation between these variables. However, the effect size of this potential relationship was small, in keeping with the limited predictive power of polygenic scores. We also explored a frequentist linear mixed modelling approach that allowed for the control of kinship by providing a participant relatedness matrix via the *coxme* pacakge (Therneau, 2022). However, this approach did not produce meaningfully different results (where applicable), and was not easily extendable to multivariate models.

**Table S1: Comparison of children retained versus those lost to follow up**

| **Characteristic** | **Lost to follow up** N = 586^1^ | **Retained** N = 1005^1^ | **p-value**^2^ | **q-value**^3^ |
| --- | --- | --- | --- | --- |
| Child Age | 11.68 (2.48) | 11.19 (2.37) | <0.001 | **0.001** |
| HSC (total) | 5.01 (1.02) | 5.05 (1.02) | 0.63 | 0.79 |
| EOE | 4.73 (1.38) | 4.74 (1.40) | 0.91 | 0.91 |
| LST | 4.96 (1.65) | 5.03 (1.61) | 0.50 | 0.79 |
| AES | 5.39 (1.09) | 5.46 (1.12) | 0.30 | 0.71 |
| PTSD | 0.95 (0.71) | 0.91 (0.73) | 0.17 | 0.71 |
| Depression | 0.84 (0.71) | 0.81 (0.70) | 0.51 | 0.79 |
| Anxiety | 1.02 (0.47) | 1.03 (0.44) | 0.89 | 0.91 |
| Externalising behaviour (caregiver-report) | 0.65 (0.23) | 0.66 (0.22) | 0.49 | 0.79 |
| Log cortisol (corrected) | 0.02 (0.44) | -0.01 (0.44) | 0.28 | 0.71 |
| Log testosterone (corrected) | 0.03 (0.59) | -0.02 (0.51) | 0.016 | 0.19 |
| Log DHEA (corrected) | 0.02 (0.35) | -0.01 (0.35) | 0.13 | 0.71 |
| Neuroticism (PGS001996) | 0.61 (0.52) | 0.59 (0.52) | 0.66 | 0.79 |
| Neuroticism (PGS002213) | 0.38 (0.54) | 0.37 (0.56) | 0.64 | 0.79 |
| Neuroticism (PGS002342) | 0.30 (0.51) | 0.27 (0.51) | 0.31 | 0.71 |
| Neuroticism (PGS002659) | 0.01 (0.12) | 0.01 (0.12) | 0.54 | 0.79 |
| Neuroticism (PGS002708) | 0.20 (0.55) | 0.16 (0.57) | 0.28 | 0.71 |
| Sensitivity/hurt feelings (PGS001016) | 0.08 (0.22) | 0.08 (0.22) | 0.79 | 0.86 |
| SESA | 0.0007 (0.0009) | 0.0007 (0.0009) | 0.33 | 0.71 |
| Sensitivity (GWAS) | 0.0029 (0.0022) | 0.0029 (0.0022) | 0.69 | 0.79 |
| Extraversion (GPC) | -0.0001 (0.0001) | -0.0001 (0.0001) | 0.20 | 0.71 |
| Neuroticism (GPC) | 0.0004 (0.0001) | 0.0004 (0.0001) | 0.63 | 0.79 |
| Sensitivity (candidate gene) | 8.51 (2.16) | 8.77 (2.21) | 0.068 | 0.54 |
| Sex |  |  | 0.35 | 0.71 |
| *Female* | 299 (51%) | 537 (53%) |  |  |
| *Male* | 287 (49%) | 468 (47%) |  |  |
| ^1^Mean (SD); n (%) | | | | |
| ^2^Wilcoxon rank sum test; Pearson's Chi-squared test | | | | |
| ^3^False discovery rate correction for multiple testing | | | | |

**Table S2: Summary of pre-determined polygenic scores used**

| Polygenic Score ID & Name | PGS Publication ID (PGP) | Reported Trait | Number of Variants | Coverage (%) |
| --- | --- | --- | --- | --- |
| PGS001996 (portability-PLR_neuroticism) | PGP000263 \|Privé F et al. Am J Hum Genet (2022) | Neuroticism score | 54,715 | 100.00 |
| PGS002213 (portability-ldpred2_neuroticism) | PGP000263 \|Privé F et al. Am J Hum Genet (2022) | Neuroticism score | 950,183 | 100.00 |
| PGS002342 (mental_NEUROTICISM.BOLT-LMM) | PGP000332 \|Weissbrod O et al. Nat Genet (2022) | Neuroticism | 1,109,311 | 99.98 |
| PGS002659 (mental_NEUROTICISM.PolyFun-pred) | PGP000332 \|Weissbrod O et al. Nat Genet (2022) | Neuroticism | 546,323 | 79.07 |
| PGS002708 (mental_NEUROTICISM.SBayesR) | PGP000332 \|Weissbrod O et al. Nat Genet (2022) | Neuroticism | 989,963 | 99.99 |
| PGS001016 (GBE_BIN1950) | PGP000244 \|Tanigawa Y et al. PLoS Genet (2022) | Sensitivity / hurt feelings | 7,922 | 93.22 |

**Table S3: Candidate gene variants used for manual PGS**

| SNP rsID | Nearest gene | Effect allele | Other allele | Example reference |
| --- | --- | --- | --- | --- |
| rs6265 | BDNF | A | G | Drury et al., 2012 |
| rs6313 | HTR2A | T | C | Jokela et al., 2007 |
| rs5522 | NR3C2 | C (Valine) | T | Bogdan et al., 2012 |
| rs4680 | COMT | G (Valine) | A | Van IJzendoorn et al, 2008 |
| rs6330 | NGF | T | C | Lester et al., 2012 |
| rs1799971 | OPRM1 | A | G | Troisi et al., 2012 |
| rs1800497 | DRD2 | T | C | Propper et al., 2008 |
| rs1360780 | FKBP5 | T | C | Binder et al., 2008 |
| rs110402 | CRHR1 | A | G | De Young et al., 2011 |
| rs4570625 | TPH2 | T | G | Forssman et al., 2014 |
| rs1488467 | OXTR | C | G | Johansson et al., 2012 |
| rs1800532 | TPH1 | T | G | Keltikangas-Jarvinen et al., 2007 |
| rs53576 | OXTR | T | C | Sturge-Apple et al., 2012 |

**Table S4: Descriptive statistics and sex comparison for the study sample**

|  | **Wave 1** | | | | **Wave 2** | | | |
| --- | --- | --- | --- | --- | --- | --- | --- | --- |
| **Variable** | **Females** N = 836^1^ | **Males** N = 755^1^ | **p-value**^2^ | **q-value**^3^ | **Females** N = 535^1^ | **Males** N = 465^1^ | **p-value**^2^ | **q-value**^3^ |
| Child Age | 11.32 (2.48) | 11.43 (2.35) | 0.18 | 0.22 | 12.30 (2.50) | 12.04 (2.21) | 0.24 | 0.27 |
| BMI | 18.2 (3.6) | 17.7 (3.5) | 0.032 | **0.042** | 18.9 (3.8) | 18.1 (3.0) | <0.001 | **<0.001** |
| War exposure | 9.0 (5.2) | 9.7 (5.5) | 0.026 | **0.039** | 9.0 (5.2) | 9.7 (5.5) | 0.026 | **0.039** |
| HSC (total) | 5.16 (0.99) | 4.89 (1.03) | <0.001 | **<0.001** | 4.91 (0.95) | 4.62 (0.94) | <0.001 | **<0.001** |
| HSC (EOE) | 4.83 (1.40) | 4.64 (1.38) | 0.005 | **0.009** | 4.50 (1.40) | 4.27 (1.46) | 0.019 | **0.033** |
| HSC (LST) | 5.20 (1.52) | 4.79 (1.70) | <0.001 | **<0.001** | 5.17 (1.55) | 4.45 (1.68) | <0.001 | **<0.001** |
| HSC (AES) | 5.55 (1.04) | 5.30 (1.16) | <0.001 | **<0.001** | 5.24 (1.19) | 5.17 (1.15) | 0.21 | 0.27 |
| PTSD | 0.95 (0.76) | 0.89 (0.68) | 0.31 | 0.31 | 0.63 (0.76) | 0.63 (0.78) | 0.66 | 0.66 |
| Depression | 0.85 (0.72) | 0.80 (0.68) | 0.30 | 0.31 | 0.67 (0.72) | 0.60 (0.65) | 0.23 | 0.27 |
| Anxiety | 1.10 (0.43) | 0.95 (0.45) | <0.001 | **<0.001** | 0.98 (0.44) | 0.79 (0.48) | <0.001 | **<0.001** |
| Externalising behaviour (caregiver report) | 0.62 (0.21) | 0.69 (0.23) | <0.001 | **<0.001** | 0.60 (0.24) | 0.71 (0.25) | <0.001 | **<0.001** |
| Ever smoked |  |  | <0.001 | **<0.001** |  |  | <0.001 | **<0.001** |
| *No* | 832 (99%) | 737 (98%) |  |  | 535 (99%) | 447 (96%) |  |  |
| *Yes* | 2 (0.2%) | 18 (2%) |  |  | 2 (1%) | 21 (4%) |  |  |
| ^1^Mean (SD); n (%) | | | | | | | | |
| ^2^Wilcoxon rank sum test; Pearson's Chi-squared test | | | | | | | | |
| ^3^False discovery rate correction for multiple testing | | | | | | | | |

**Table S5: Descriptive statistics and sex comparison for hair hormone variables**

|  | **Wave 1** | | | | **Wave 2** | | | |
| --- | --- | --- | --- | --- | --- | --- | --- | --- |
| **Characteristic** | **Female** N = 836^1^ | **Male** N = 755^1^ | **p-value**^2^ | **q-value**^3^ | **Female** N = 535^1^ | **Male** N = 465^1^ | **p-value**^2^ | **q-value**^3^ |
| Log cortisol (corrected) | 0.12 (0.43) | -0.13 (0.41) | <0.001 | **<0.001** | 0.09 (0.42) | -0.12 (0.35) | <0.001 | **<0.001** |
| Log testosterone (corrected) | -0.10 (0.62) | 0.11 (0.42) | <0.001 | **<0.001** | -0.09 (0.44) | 0.12 (0.36) | <0.001 | **<0.001** |
| Log DHEA (corrected) | -0.18 (0.34) | 0.20 (0.23) | <0.001 | **<0.001** | -0.13 (0.28) | 0.18 (0.20) | <0.001 | **<0.001** |
| Frequent hair alterations |  |  | <0.001 | **<0.001** |  |  | <0.001 | **<0.001** |
| *No* | 484 (58%) | 672 (89%) |  |  | 338 (63%) | 426 (91%) |  |  |
| *Yes* | 350 (42%) | 83 (11%) |  |  | 199 (37%) | 41 (9%) |  |  |
| Frequency of hair washing |  |  | <0.001 | **<0.001** |  |  | <0.001 | **<0.001** |
| *2-4 times per week* | 553 (66%) | 416 (55%) |  |  | 324 (60%) | 234 (50%) |  |  |
| *5-7 times per week* | 215 (26%) | 281 (37%) |  |  | 182 (34%) | 219 (47%) |  |  |
| *Once per week* | 66 (8%) | 58 (8%) |  |  | 30 (6%) | 15 (3%) |  |  |
| ^1^Mean (SD); n (%) | | | | | | | | |
| ^2^Wilcoxon rank sum test; Pearson's Chi-squared test | | | | | | | | |
| ^3^False discovery rate correction for multiple testing | | | | | | | | |

**Table S6: Average, z-standardised polygenic scores**

| **Characteristic** | **Female** N = 836^1^ | **Male** N = 755^1^ | **p-value**^2^ | **q-value**^3^ |
| --- | --- | --- | --- | --- |
| Neuroticism (PGS001996) | 0.06 (0.99) | -0.06 (1.00) | 0.055 | 0.19 |
| Neuroticism (PGS002213) | 0.05 (0.99) | -0.06 (1.01) | 0.067 | 0.19 |
| Neuroticism (PGS002342) | 0.05 (0.98) | -0.06 (1.01) | 0.10 | 0.19 |
| Neuroticism (PGS002659) | 0.05 (0.99) | -0.05 (1.01) | 0.085 | 0.19 |
| Neuroticism (PGS002708) | 0.05 (0.99) | -0.06 (1.01) | 0.10 | 0.19 |
| Neuroticism (GPC) | 0.04 (0.97) | -0.04 (1.03) | 0.21 | 0.29 |
| Extraversion (GPC) | -0.01 (1.00) | 0.01 (1.00) | 0.51 | 0.62 |
| Sensitivity/hurt feelings (PGS001016) | 0.04 (1.02) | -0.04 (0.97) | 0.15 | 0.24 |
| Sensitivity to environmental stress and adversity | 0.05 (1.01) | -0.06 (0.98) | 0.030 | 0.19 |
| Sensitivity (GWAS) | 0.01 (1.00) | -0.01 (1.00) | 0.64 | 0.70 |
| Sensitivity (candidate gene) | 0.00 (1.01) | 0.00 (0.99) | 0.97 | 0.97 |
| ^1^Mean (SD) | | | | |
| ^2^Wilcoxon rank sum test | | | | |
| ^3^False discovery rate correction for multiple testing | | | | |

**Table S7: Zero-order correlation coefficients between study variables**

|  | HSC | EOE | LST | AES | Log cortisol | Log testosterone | Log DHEA | PGS001996 | PGS002213 | PGS002342 | PGS002659 | PGS002708 | PGS001016 | SESA | Sensitivity (GWAS) | Extraversion (GPC) | Neuroticism (GPC) | Sensitivity (candidate gene) |
| --- | --- | --- | --- | --- | --- | --- | --- | --- | --- | --- | --- | --- | --- | --- | --- | --- | --- | --- |
| HSC |  |  |  |  |  |  |  |  |  |  |  |  |  |  |  |  |  |  |
| EOE | 0.85*** |  |  |  |  |  |  |  |  |  |  |  |  |  |  |  |  |  |
| LST | 0.73*** | 0.43*** |  |  |  |  |  |  |  |  |  |  |  |  |  |  |  |  |
| AES | 0.63*** | 0.29*** | 0.25*** |  |  |  |  |  |  |  |  |  |  |  |  |  |  |  |
| Log cortisol | 0.10*** | 0.07** | 0.05 | 0.09*** |  |  |  |  |  |  |  |  |  |  |  |  |  |  |
| Log testosterone | 0.02 | 0.04 | -0.03 | 0.02 | 0.48*** |  |  |  |  |  |  |  |  |  |  |  |  |  |
| Log DHEA | -0.04 | 0.01 | -0.08** | -0.03 | 0.05* | 0.48*** |  |  |  |  |  |  |  |  |  |  |  |  |
| PGS001996 | -0.01 | -0.02 | 0.03 | -0.01 | 0.01 | 0.02 | 0.02 |  |  |  |  |  |  |  |  |  |  |  |
| PGS002213 | -0.01 | -0.04 | 0.03 | -0.01 | 0.00 | 0.01 | 0.02 | 0.87*** |  |  |  |  |  |  |  |  |  |  |
| PGS002342 | -0.03 | -0.05 | 0.01 | -0.02 | 0.01 | 0.02 | 0.02 | 0.69*** | 0.83*** |  |  |  |  |  |  |  |  |  |
| PGS002659 | -0.01 | -0.02 | 0.01 | -0.01 | -0.01 | -0.01 | 0.00 | 0.65*** | 0.67*** | 0.67*** |  |  |  |  |  |  |  |  |
| PGS002708 | -0.02 | -0.04 | 0.01 | -0.01 | 0.01 | 0.03 | 0.03 | 0.67*** | 0.80*** | 0.95*** | 0.68*** |  |  |  |  |  |  |  |
| PGS001016 | 0.01 | 0.01 | 0.00 | 0.02 | 0.00 | -0.01 | 0.01 | 0.31*** | 0.31*** | 0.34*** | 0.29*** | 0.30*** |  |  |  |  |  |  |
| SESA | 0.03 | 0.02 | 0.03 | 0.03 | -0.01 | -0.03 | -0.01 | 0.19*** | 0.17*** | 0.13*** | 0.23*** | 0.12*** | 0.17*** |  |  |  |  |  |
| Sensitivity (GWAS) | -0.05 | -0.04 | -0.02 | -0.05 | -0.01 | -0.03 | 0.00 | 0.03 | 0.05 | 0.07** | 0.02 | 0.05 | 0.02 | 0.00 |  |  |  |  |
| Extraversion (GPC) | 0.03 | 0.01 | 0.02 | 0.03 | -0.01 | -0.02 | 0.01 | -0.02 | -0.02 | -0.01 | 0.00 | 0.00 | 0.03 | -0.02 | -0.05 |  |  |  |
| Neuroticism (GPC) | 0.09** | 0.08** | 0.06* | 0.06* | 0.00 | -0.01 | -0.02 | 0.03 | 0.04 | 0.01 | 0.01 | 0.01 | 0.03 | -0.02 | 0.03 | -0.10*** |  |  |
| Sensitivity (candidate gene) | -0.03 | -0.06* | 0.01 | 0.00 | -0.01 | 0.01 | -0.02 | 0.02 | 0.00 | -0.01 | 0.01 | -0.02 | 0.01 | -0.08** | -0.02 | -0.01 | 0.00 |  |

**Table S8: Intraclass correlation coefficients**

| Variable | ICC^a^ |
| --- | --- |
| HSC (total) | 0.09 |
| EOE | 0.06 |
| LST | 0.07 |
| AES | 0.06 |
| PTSD | 0.13 |
| Depression | 0.26 |
| Anxiety | 0.25 |
| Externalising behaviour | 0.35 |
| Log cortisol | 0.44 |
| Log testosterone | 0.51 |
| Log DHEA | 0.63 |
| ^a^ICC = Intraclass correlation coefficient; EOE = Ease of Excitation; LST = Low Sensory Threshold; AES = Aesthetic Sensitivity | |

**Table S9: Multivariate regression model of predictors of sensitivity subscales**

| Coefficient | Estimate | Error | Lower 95% CI^a^ | Upper 95% CI^a^ |
| --- | --- | --- | --- | --- |
| **Ease of Excitation** |  |  |  |  |
| Intercept | 3.926 | 0.432 | **3.078** | **4.784** |
| Wave | -0.525 | 0.074 | **-0.668** | **-0.381** |
| Sex (female) | 0.284 | 0.097 | **0.091** | **0.475** |
| Age | 0.024 | 0.019 | -0.015 | 0.063 |
| War exposure | 0.020 | 0.007 | **0.007** | **0.034** |
| Log cortisol | 0.032 | 0.111 | -0.186 | 0.240 |
| Log testosterone | 0.091 | 0.116 | -0.139 | 0.321 |
| Log DHEA | 0.110 | 0.155 | -0.192 | 0.423 |
| PGS001996 | -0.054 | 0.078 | -0.206 | 0.100 |
| PGS002213 | -0.063 | 0.099 | -0.261 | 0.127 |
| PGS002342 | -0.021 | 0.125 | -0.271 | 0.220 |
| PGS002659 | -0.019 | 0.052 | -0.122 | 0.083 |
| PGS002708 | 0.127 | 0.116 | -0.101 | 0.352 |
| PGS001016 | -0.048 | 0.039 | -0.124 | 0.028 |
| SESA | 0.023 | 0.039 | -0.055 | 0.099 |
| Sensitivity (GWAS) | -0.032 | 0.036 | -0.102 | 0.040 |
| Neuroticism (GPC) | 0.048 | 0.037 | -0.024 | 0.120 |
| Extraversion (GPC) | 0.042 | 0.036 | -0.028 | 0.113 |
| Sensitivity (CG) | -0.024 | 0.037 | -0.095 | 0.047 |
| **Low Sensory Threshold** |  |  |  |  |
| Intercept | 3.959 | 0.501 | **2.946** | **4.925** |
| Wave | -0.175 | 0.086 | **-0.343** | **-0.004** |
| Sex (female) | 0.793 | 0.112 | **0.565** | **1.012** |
| Age | -0.032 | 0.022 | -0.075 | 0.010 |
| War exposure | 0.016 | 0.008 | **0.001** | **0.032** |
| Log cortisol | -0.113 | 0.128 | -0.367 | 0.137 |
| Log testosterone | 0.029 | 0.134 | -0.233 | 0.292 |
| Log DHEA | -0.035 | 0.181 | -0.394 | 0.316 |
| PGS001996 | -0.056 | 0.090 | -0.233 | 0.119 |
| PGS002213 | 0.073 | 0.114 | -0.148 | 0.298 |
| PGS002342 | -0.056 | 0.141 | -0.333 | 0.227 |
| PGS002659 | 0.025 | 0.060 | -0.095 | 0.143 |
| PGS002708 | 0.028 | 0.131 | -0.230 | 0.286 |
| PGS001016 | -0.032 | 0.045 | -0.120 | 0.055 |
| SESA | -0.017 | 0.045 | -0.104 | 0.071 |
| Sensitivity (GWAS) | -0.028 | 0.043 | -0.111 | 0.056 |
| Neuroticism (GPC) | 0.010 | 0.043 | -0.074 | 0.094 |
| Extraversion (GPC) | 0.070 | 0.042 | -0.011 | 0.151 |
| Sensitivity (CG) | 0.016 | 0.042 | -0.064 | 0.099 |
| **Aesthetic Sensitivity** |  |  |  |  |
| Intercept | 4.840 | 0.339 | **4.184** | **5.515** |
| Wave | -0.300 | 0.060 | **-0.419** | **-0.181** |
| Sex (female) | 0.230 | 0.077 | **0.081** | **0.384** |
| Age | 0.012 | 0.015 | -0.017 | 0.042 |
| War exposure | 0.001 | 0.006 | -0.010 | 0.012 |
| Log cortisol | 0.033 | 0.087 | -0.138 | 0.202 |
| Log testosterone | 0.130 | 0.093 | -0.053 | 0.314 |
| Log DHEA | 0.091 | 0.126 | -0.161 | 0.337 |
| PGS001996 | -0.033 | 0.061 | -0.153 | 0.087 |
| PGS002213 | -0.045 | 0.077 | -0.196 | 0.103 |
| PGS002342 | -0.038 | 0.100 | -0.236 | 0.157 |
| PGS002659 | 0.020 | 0.042 | -0.060 | 0.103 |
| PGS002708 | 0.002 | 0.092 | -0.178 | 0.184 |
| PGS001016 | 0.025 | 0.031 | -0.035 | 0.084 |
| SESA | 0.031 | 0.030 | -0.028 | 0.089 |
| Sensitivity (GWAS) | -0.005 | 0.030 | -0.063 | 0.054 |
| Neuroticism (GPC) | 0.024 | 0.029 | -0.033 | 0.082 |
| Extraversion (GPC) | 0.060 | 0.029 | **0.003** | **0.117** |
| Sensitivity (CG) | 0.035 | 0.029 | -0.022 | 0.091 |
| ^a^CI = Credible Interval; CG = candidate gene; SESA = Sensitivity to environmental stress and adversity; GPC = Genetics of Personality Consortium; PGS = polygenic score. Bolded intervals are those not including 0 | | | | |

**Table S10: Association of self-reported sensitivity subscales with mental health outcomes, using a multivariate linear mixed model.**

| Coefficient | Estimate | Error | Lower 95% CI^a^ | Upper 95% CI^a^ |
| --- | --- | --- | --- | --- |
| **Anxiety** |  |  |  |  |
| Intercept | -0.695 | 0.326 | **-1.330** | **-0.044** |
| Wave | 0.129 | 0.051 | **0.028** | **0.228** |
| Sex (female) | 0.315 | 0.070 | **0.177** | **0.449** |
| EOE | 0.110 | 0.020 | **0.071** | **0.148** |
| LST | 0.057 | 0.017 | **0.024** | **0.090** |
| AES | 0.046 | 0.024 | -0.002 | 0.091 |
| Age | -0.042 | 0.013 | **-0.068** | **-0.015** |
| War exposure | 0.014 | 0.005 | **0.004** | **0.024** |
| Log cortisol | 0.143 | 0.079 | -0.013 | 0.296 |
| Log testosterone | 0.014 | 0.082 | -0.145 | 0.176 |
| Log DHEA | 0.219 | 0.108 | **0.009** | **0.431** |
| PGS001996 | 0.039 | 0.055 | -0.070 | 0.147 |
| PGS002213 | -0.028 | 0.069 | -0.163 | 0.109 |
| PGS002342 | 0.070 | 0.088 | -0.103 | 0.243 |
| PGS002659 | -0.018 | 0.036 | -0.089 | 0.054 |
| PGS002708 | -0.002 | 0.081 | -0.157 | 0.155 |
| PGS001016 | -0.008 | 0.027 | -0.063 | 0.045 |
| SESA | -0.006 | 0.027 | -0.060 | 0.047 |
| Sensitivity (GWAS) | -0.001 | 0.026 | -0.053 | 0.049 |
| Neuroticism (GPC) | 0.016 | 0.026 | -0.036 | 0.068 |
| Extraversion (GPC) | 0.012 | 0.026 | -0.038 | 0.062 |
| Sensitivity (CG) | -0.036 | 0.026 | -0.086 | 0.015 |
| **PTSD** |  |  |  |  |
| Intercept | -0.754 | 0.326 | **-1.389** | **-0.110** |
| Wave | 0.037 | 0.053 | -0.068 | 0.141 |
| Sex (female) | -0.004 | 0.070 | -0.144 | 0.131 |
| EOE | 0.135 | 0.020 | **0.095** | **0.174** |
| LST | 0.020 | 0.017 | -0.013 | 0.053 |
| AES | 0.035 | 0.024 | -0.012 | 0.082 |
| Age | 0.025 | 0.014 | -0.002 | 0.052 |
| War exposure | 0.025 | 0.005 | **0.015** | **0.035** |
| Log cortisol | 0.068 | 0.077 | -0.080 | 0.219 |
| Log testosterone | 0.106 | 0.081 | -0.050 | 0.265 |
| Log DHEA | 0.047 | 0.111 | -0.172 | 0.263 |
| PGS001996 | 0.063 | 0.056 | -0.046 | 0.174 |
| PGS002213 | 0.044 | 0.070 | -0.093 | 0.180 |
| PGS002342 | 0.122 | 0.089 | -0.054 | 0.298 |
| PGS002659 | -0.095 | 0.037 | **-0.166** | **-0.021** |
| PGS002708 | -0.118 | 0.082 | -0.280 | 0.043 |
| PGS001016 | -0.038 | 0.028 | -0.093 | 0.017 |
| SESA | 0.023 | 0.027 | -0.029 | 0.076 |
| Sensitivity (GWAS) | -0.032 | 0.026 | -0.083 | 0.019 |
| Neuroticism (GPC) | 0.044 | 0.026 | -0.008 | 0.095 |
| Extraversion (GPC) | -0.012 | 0.026 | -0.063 | 0.039 |
| Sensitivity (CG) | -0.003 | 0.026 | -0.054 | 0.048 |
| **Depression** |  |  |  |  |
| Intercept | -1.336 | 0.320 | **-1.964** | **-0.708** |
| Wave | 0.054 | 0.049 | -0.042 | 0.150 |
| Sex (female) | 0.161 | 0.069 | **0.027** | **0.296** |
| EOE | 0.109 | 0.019 | **0.072** | **0.148** |
| LST | 0.039 | 0.016 | **0.006** | **0.071** |
| AES | -0.013 | 0.022 | -0.057 | 0.032 |
| Age | 0.055 | 0.013 | **0.028** | **0.081** |
| War exposure | 0.022 | 0.005 | **0.012** | **0.031** |
| Log cortisol | 0.102 | 0.077 | -0.051 | 0.253 |
| Log testosterone | -0.116 | 0.081 | -0.273 | 0.050 |
| Log DHEA | 0.365 | 0.109 | **0.149** | **0.582** |
| PGS001996 | -0.020 | 0.055 | -0.131 | 0.086 |
| PGS002213 | 0.051 | 0.071 | -0.086 | 0.189 |
| PGS002342 | 0.083 | 0.089 | -0.091 | 0.259 |
| PGS002659 | -0.020 | 0.037 | -0.093 | 0.052 |
| PGS002708 | -0.027 | 0.081 | -0.187 | 0.131 |
| PGS001016 | 0.009 | 0.028 | -0.045 | 0.062 |
| SESA | -0.007 | 0.028 | -0.062 | 0.047 |
| Sensitivity (GWAS) | 0.012 | 0.026 | -0.040 | 0.062 |
| Neuroticism (GPC) | 0.019 | 0.026 | -0.032 | 0.072 |
| Extraversion (GPC) | -0.033 | 0.026 | -0.083 | 0.019 |
| Sensitivity (CG) | 0.010 | 0.026 | -0.041 | 0.062 |
| ^a^CI = Credible Interval; CG = candidate gene; SESA = Sensitivity to environmental stress and adversity; GPC = Genetics of Personality Consortium; PGS = polygenic score. Bolded intervals are those not including 0. | | | | |

**Table S11: Association of self-reported sensitivity subscales with caregiver-reported externalising behaviour**

| Coefficient | Estimate | Error | Lower 95% CI^a^ | Upper 95% CI^a^ |
| --- | --- | --- | --- | --- |
| Intercept | 0.787 | 0.078 | **0.632** | **0.939** |
| Wave | 0.022 | 0.012 | -0.001 | 0.046 |
| Sex (female) | -0.103 | 0.017 | **-0.136** | **-0.069** |
| EOE | 0.007 | 0.005 | -0.002 | 0.016 |
| LST | -0.003 | 0.004 | -0.010 | 0.005 |
| AES | 0.007 | 0.005 | -0.003 | 0.018 |
| Age | -0.013 | 0.003 | **-0.019** | **-0.007** |
| War exposure | 0.004 | 0.001 | **0.002** | **0.007** |
| Log cortisol | 0.040 | 0.018 | **0.005** | **0.075** |
| Log testosterone | 0.002 | 0.019 | -0.034 | 0.040 |
| Log DHEA | -0.008 | 0.026 | -0.060 | 0.043 |
| PGS001996 | 0.031 | 0.013 | **0.005** | **0.056** |
| PGS002213 | -0.018 | 0.017 | -0.050 | 0.014 |
| PGS002342 | 0.020 | 0.021 | -0.021 | 0.061 |
| PGS002659 | -0.010 | 0.009 | -0.027 | 0.008 |
| PGS002708 | -0.014 | 0.019 | -0.052 | 0.024 |
| PGS001016 | -0.002 | 0.007 | -0.015 | 0.010 |
| SESA | 0.003 | 0.007 | -0.010 | 0.016 |
| Sensitivity (GWAS) | -0.000 | 0.006 | -0.012 | 0.012 |
| Neuroticism (GPC) | 0.006 | 0.006 | -0.006 | 0.019 |
| Extraversion (GPC) | -0.010 | 0.006 | -0.022 | 0.002 |
| Sensitivity (CG) | 0.001 | 0.006 | -0.011 | 0.013 |
| ^a^CI = Credible Interval; CG = candidate gene; SESA = Sensitivity to environmental stress and adversity; GPC = Genetics of Personality Consortium; PGS = polygenic score. Bolded intervals are those not including 0. | | | | |
